# Functional soft palate reconstruction

**DOI:** 10.1016/j.jpra.2024.11.016

**Published:** 2024-12-02

**Authors:** Sofia Oetliker-Contin, Tarek Ismail, Rik Osinga, Maximilian Burger, Jens Jakscha, Claude Fischer, Laurent Muller, Carlo M. Oranges, Dirk J. Schaefer

**Affiliations:** a Department of Plastic, Reconstructive, Aesthetic, and Hand Surgery, University Hospital Basel, Basel, Switzerland; b Center for Musculoskeletal Infections (ZMSI), University Hospital Basel, Switzerland; c Canniesburn Plastic Surgery Unit, Glasgow Royal Infirmary, 84 Castle Street, Glasgow, UK; d Praxis beim Merian Iselin, Thannerstrasse 80, Basel, Switzerland; e REHAB Basel, Clinic for Neurorehabilitation and Paraplegiology, Basel, Switzerland; f Department of Otolaryngology, Head and Neck Surgery, University Hospital Basel, Basel, Switzerland; g HNO Zentrum Thun AG, Thun, Switzerland; h Department of Plastic, Reconstructive and Aesthetic Surgery, Geneva University Hospitals, Geneva, Switzerland

**Keywords:** Soft palate reconstruction, Digastric muscle, Functional reconstruction, Oropharyngeal carcinoma

## Abstract

**Background:**

The excision of oropharyngeal carcinoma of more than 50% of the soft palate followed by static reconstruction may result in functional deficits, including velopharyngeal insufficiency, swallowing, and speech difficulties. We describe a functional soft palate reconstruction technique aimed at restoring aeromechanical and acoustic functions, enabling swallowing without nasal regurgitation and speech with low nasalance.

**Material and Methods:**

We developed a new operative technique, using muscle transfer and a free flap to create a dynamic reconstruction. To prove the distinct nerve innervation of the two digastric bellies and the feasibility of the technique, we first performed an anatomical study, and then implemented the technique in our clinic. The surgical technique included transfer of the anterior and posterior bellies of the digastric muscle in association with a folded radial forearm free flap. A retrospective analysis of patients who underwent this soft palate functional reconstruction after cancer resection between 2007 and 2017 was performed, and a subjective analysis of nasalance and swallowing was done to evaluate the functional outcomes.

**Results:**

Eight patients (six males, two females) with a mean age of 56 years (range 43–69) who were affected by oropharynx carcinoma (stage T1-3) infiltrating the soft palate were included. Analysis of the reconstruction showed that seven of the eight patients had satisfactory swallowing function, and all patients were able to speak in an understandable manner with minimal nasalance.

**Conclusions:**

Our surgical approach provided a functional reconstruction with outcomes close to normality, making it a suitable technique for patients with large soft palate defects.

## Introduction

The treatment of infiltrative oropharyngeal carcinoma involving more than 50% of the soft palate by excision may result in functional deficits such as velopharyngeal insufficiency and consequent swallowing and speech difficulties.^1^^,^^2^ Specific problems include regurgitation and formant formation, which are related to the absence of a posterior and medial shift of the soft palate required to close the epipharynx.

Static, anatomical, and microsurgical reconstruction approaches are currently used. Static reconstruction is generally performed with postpharyngeal wall augmentation using autologous or alloplastic means.^3^^,^^4^ However, this technique does not provide speech improvement; moreover, obstruction of the upper airways has been reported as a possible complication. Anatomical reconstruction is performed with posterior pharyngeal flaps and pharyngoplasty. It is indicated in cases of small defects associated with tumors staged T1-2. Results for speaking ability are reported to be moderate to poor.^5^^,^^6^ A microsurgical approach is commonly performed by using a free forearm flap with a folded patch, sometimes including the palmaris longus tendon. Sinha et al.^7^ showed in 2004 that the radial forearm free flap (RFFF) in a folded technique led to intelligible speech and oral intake in 14 of 16 patients.

These results confirmed the rationale for using a microvascular flap for soft palate reconstruction. The flap is soft, pliable, and thin and therefore suitable for inset in this anatomical area. However, this strategy does not restore velopharyngeal function.^7^, ^8^, ^9^, ^10^ As a consequence of poor functional outcomes for speech and swallowing after surgical resection and nonphysiological reconstruction, primary radiochemotherapy (RCTx) is indicated in these cases at interdisciplinary oropharyngeal cancer treatment centers.

To achieve a functional soft palate reconstruction and restore physiological conditions, attention has to be paid to velopharyngeal sphincter function (medial shift, posterior extension, and narrowing of velopharyngeal space). To reach this goal, we used a folded RFFF in combination with the transposition of the posterior digastric muscle with extension of the digastricus anterior tendon.

The digastric muscle, a member of the suprahyoid muscle group, has two independently innervated bellies^11^ and an intermediate tendon in the form of a fibrous loop that inserts on the greater horn of the hyoid bone.^12^ The overlying stylohyoid muscle encircles the intermediate tendon of the digastric muscles with its two insertion tendons at the hyoid bone. The posterior belly has its origin at the digastric ridge of the mastoid process^13^ and the anterior belly at the digastric fossa of the mandible.^12^

Using a 320-row detector computed tomography scanner, Okada et al.^13^ showed in a study that the posterior digastric muscle plays a key role at the beginning stage of the swallowing process and contracts simultaneously with the mylohyoid and stylohyoid muscles. This contraction leads to an upward movement of the hyoid bone and initiates the reflex for swallowing. The anterior belly, however, appears to have more of a supporting function by stabilizing the hyoid bone during the subsequent swallowing process, during which the hyoid bone is moved forward. Here, the geniohyoid muscle seems to be the key muscle.^14^

We aimed to develop a soft palate reconstruction technique for improved aeromechanical and acoustic functions, with the specific advantages of allowing swallowing without nasal regurgitation and speech quality without nasalance. To this end, a multispecialist approach with contributions from plastic surgery and otolaryngology, applying concepts derived from microsurgical free tissue transfer and cleft lip repair, was adopted. Evaluations of the results were conducted through independent postoperative logopedic examination.

The independent innervation of the two muscle bellies, as described in previous studies,^11^ is crucial in this functional technique. An anatomical study was carried out to better understand whether a transfer of the muscle could lead to innervation problems of the posterior venter and whether the technique was feasible.

## Material and methods

We performed a retrospective study that included all patients who underwent functional reconstruction of the soft palate with transposition of the digastric muscle and RFFF following oncological resection at the Interdisciplinary Head and Neck Cancer Center of the University Hospital of Basel between December 2007 and March 2017. All reconstructions were done by the senior author (D.J.S.). Surgical records were screened to identify eligible patients. The clinical history of the patients was then analyzed for oncological diagnosis according to TNM classification, demographic data, defect size, surgical results and complications, oncological perioperative therapy (chemotherapy, radiotherapy), and functional results (speech and swallowing functionality), as described in medical examinations, operative reports, radiological investigations, and pathological findings. Data were searched and collected by using our electronic patient recording system (ISMed, ProtecData, Boswil AG, Switzerland).

To support the anatomical basis of the technique, we additionally performed a cadaveric study with three human cadavers (2 females, 1 male) preserved by Thiel's method in order to demonstrate the anatomy of the digastric muscle and its double innervation. We measured the distances with a centimeter scale. We observed that the muscle transposition changed the movement of the digastric muscles from a former upward movement (over the inferior hyoid bone fixation, which serves as a hypomochlion for the superior anteromedial to superior posterolateral muscle vectors of the two bellies) to an inferior ipsilateral to superior contralateral muscle contraction. To our knowledge, this should narrow the pharyngeal opening during swallowing. Furthermore, we observed that the length of the transposed muscle is enough to reach the contralateral side of the soft palate.

### Surgical technique

**Figure 1:**
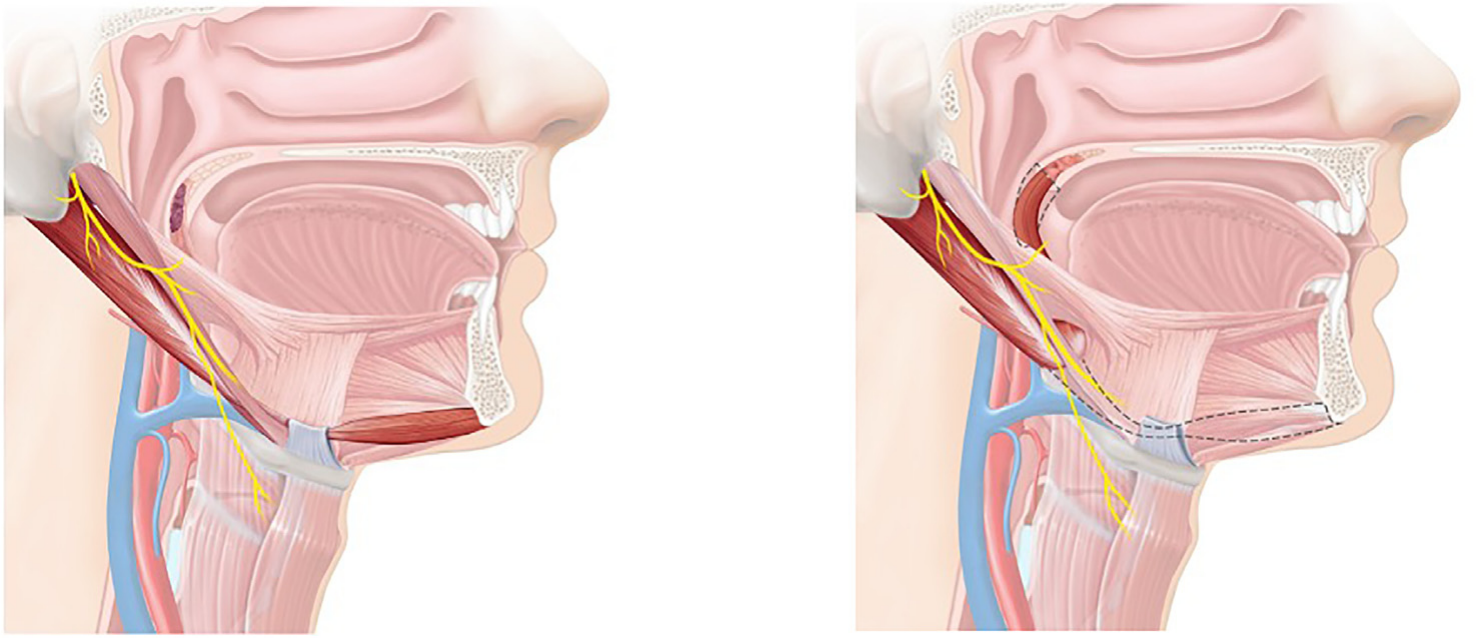
Transposition of the digastric muscle with the overlying stylohyoid muscle.

Functional muscle transfer of the posterior belly of the digastric muscle was performed in association with a reconstructive procedure that includes a folded RFFF. The innervation of the posterior belly of the digastric muscle is provided by the facial nerve. Care must be taken to keep the innervation and blood supply of the posterior belly intact; the anterior belly is denervated and functions as an extended tendon of the posterior belly (Figure 1).

**Figure 2:**
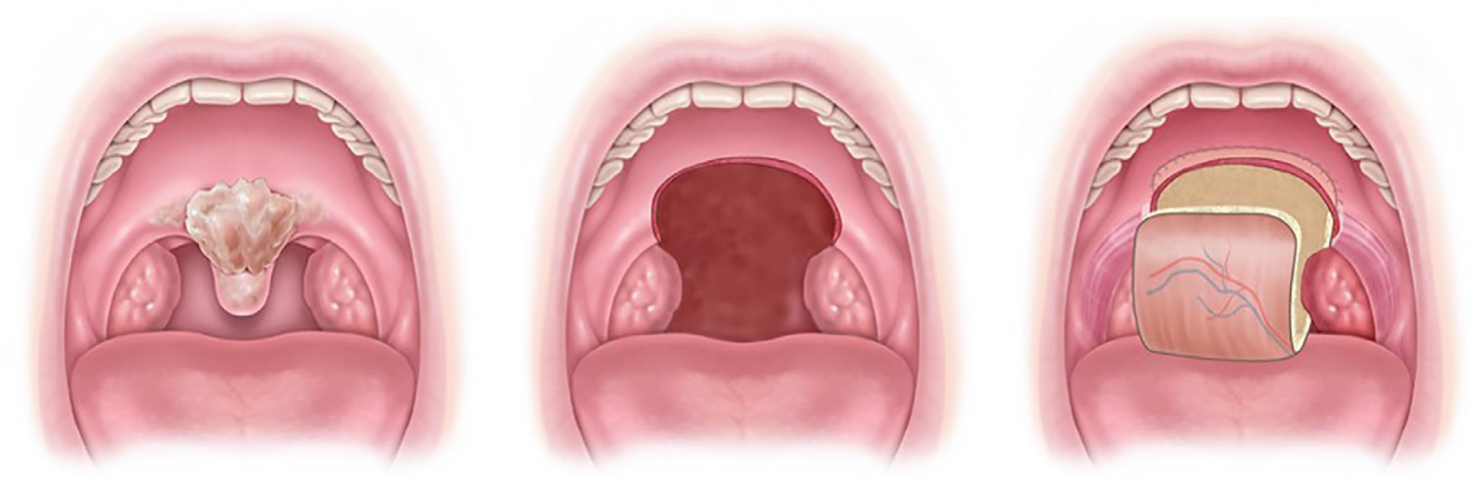
Schematic pictures of the tumor location, defect size, and posterior inset of the radial forearm free flap.

**Figure 3:**
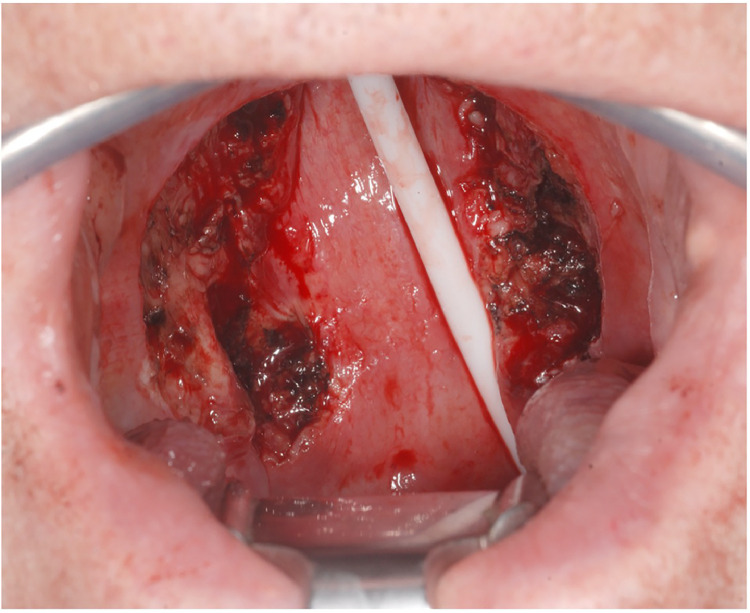
Defect after cancer resection with nasogastric tube.

Tumorectomy of the oropharynx included at least two-thirds of the soft palate in almost all patients and one half of the soft palate in one case (Figure 2, Figure 3).

**Figure 4:**
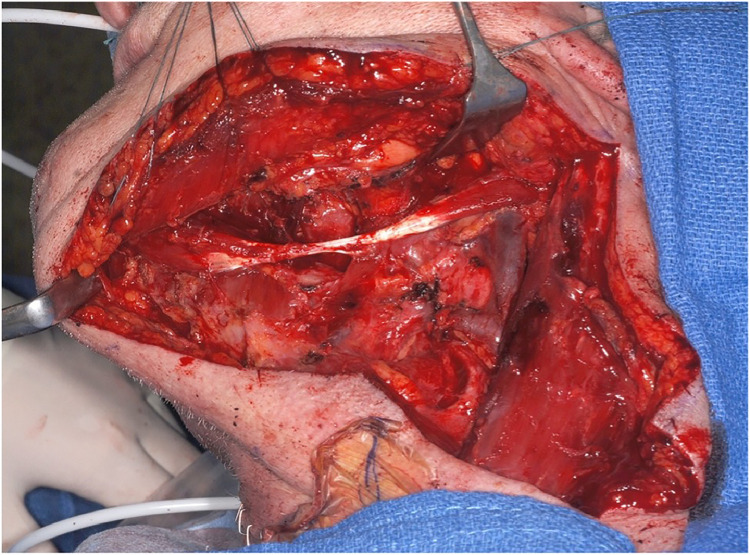
View of the digastric muscle with its two muscle bellies and the interjacent tendon in the context of the neck dissection.

Afterward, preparation of the digastric muscle was performed through an external access placed in the lateral aspect of the neck in the context of the neck dissection (Figure 4). After completion of the neck dissection, the anterior belly of the digastric muscle was separated from its origin on the mandible by using monopolar electrocautery.

**Figure 5:**
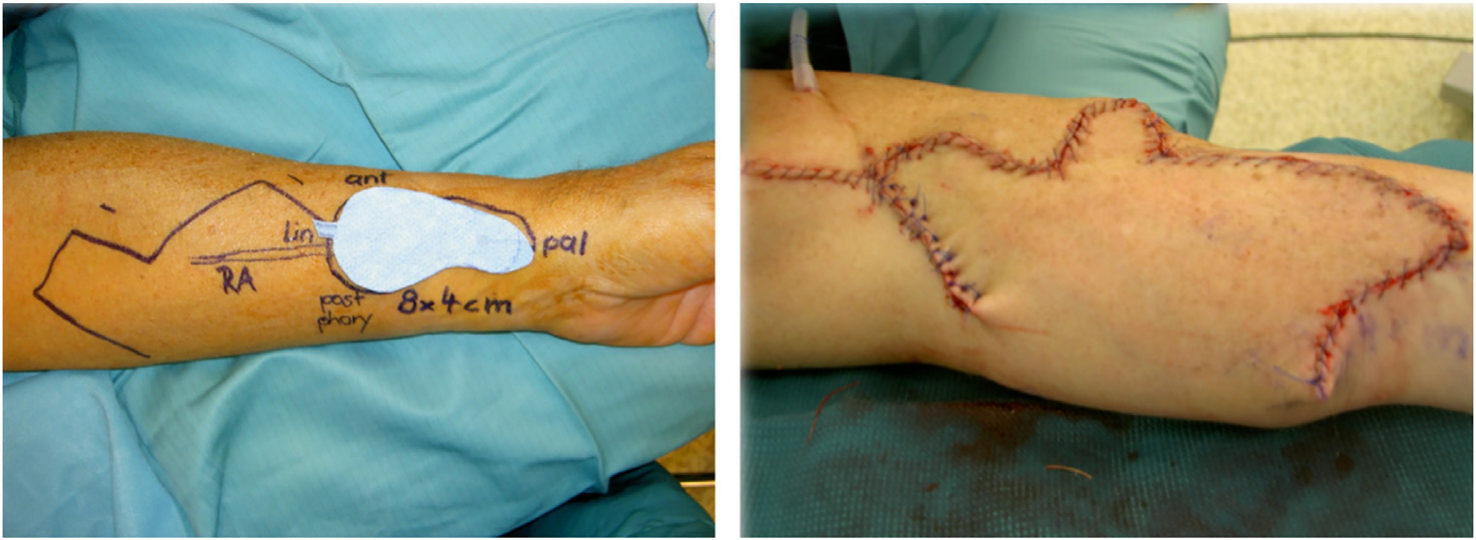
Preoperative planning of the radial forearm free flap and closure of the donor side.

The fibrous loop that keeps the intermediate tendon of the digastric muscle connected to the hyoid bone was opened to allow mobilization. The anterior belly could then be pulled through the tendons of the stylohyoid muscle to free the digastric muscle from the hyoid bone. Meanwhile, the radialis flap was harvested according to the defect template with a pedicle of about 10 cm (Figure 5).

Easy-flow drainage placement helped guide the way from external to internal through a pharyngostomy (supplementary video). The flap was transferred into the oral cavity and the pedicle flushed down the easy-flow drainage by using water and creating a vacuum with a suction drain on the external part of the drainage.

**Figure 6:**
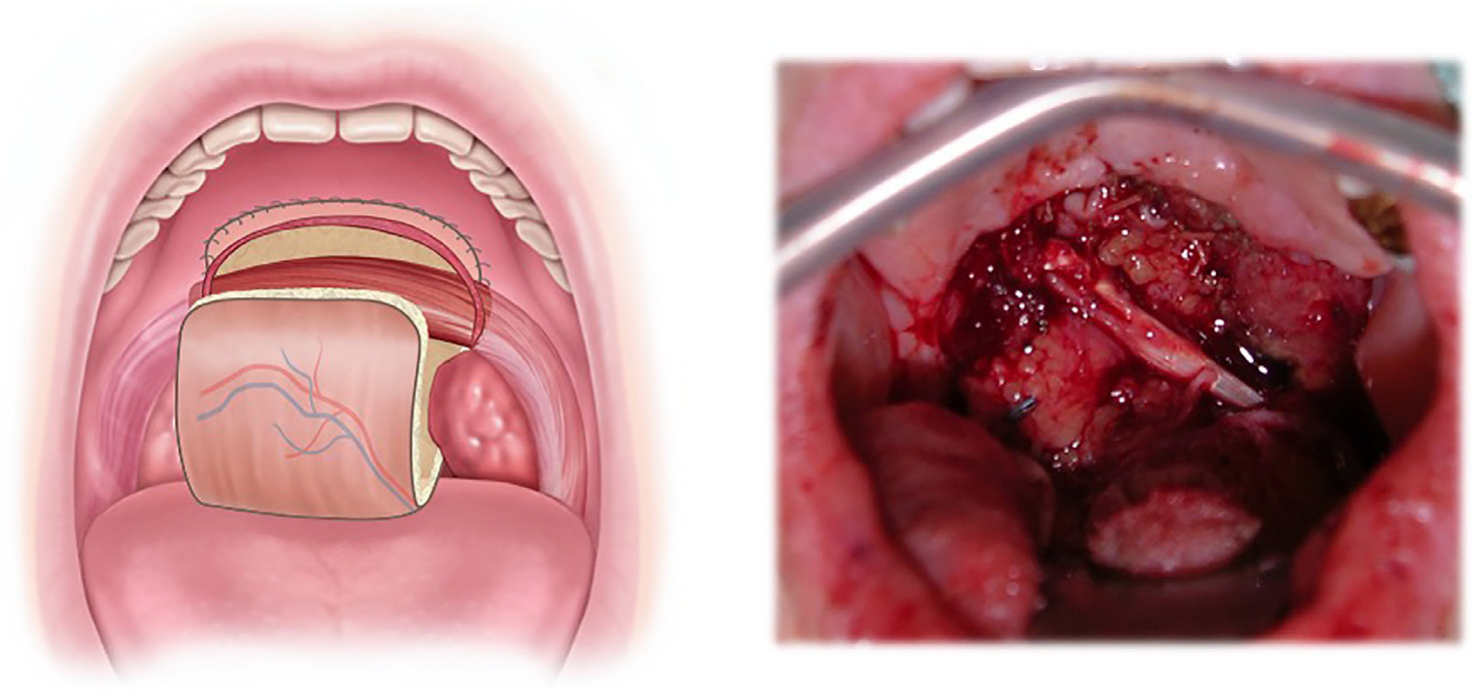
Schematic and intraoperative picture of fixed muscle showing the sandwich technique after inset of the posterior wall of the radial forearm free flap.

To cover the tumor resection area and reconstruct the nasopharyngeal and oropharyngeal tube, a neovelum was then created with a folded RFFF by first suturing the back wall to the epipharynx (Figure 6) (hard palate and remaining soft tissue) in an inside-out-fashion with 3.0 Polyglactin 910 sutures (Vicryl, Ethicon, Raritan, New Jersey, US). The pedicle of the RFFF was guided medial to the mandible in parallel and temporarily stored in the oral cavity.

The denervated anterior belly and the still innervated posterior belly of the digastric muscle were then transferred into the oral cavity to the contralateral side of the soft palate remnants, where the anterior venter tendon was sutured with 3.0 Polyglactin 910 (Vicryl, Ethicon, Raritan, New Jersey, US) under tension to help the posterior belly to pull on the velum (Figure 6).

**Figure 7:**
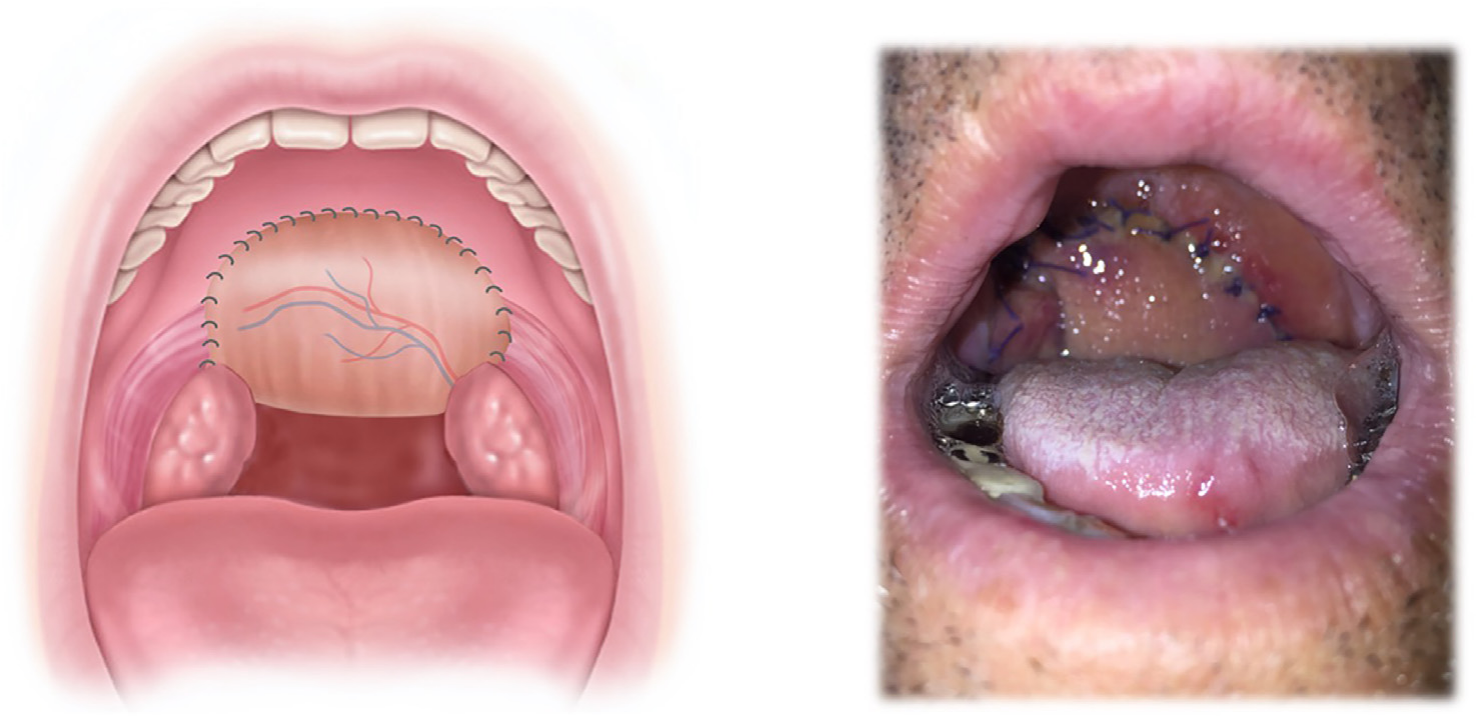
Schematic and intraoperative picture after inset of the anterior wall of the radial forearm free flap.

**Figure 8:**
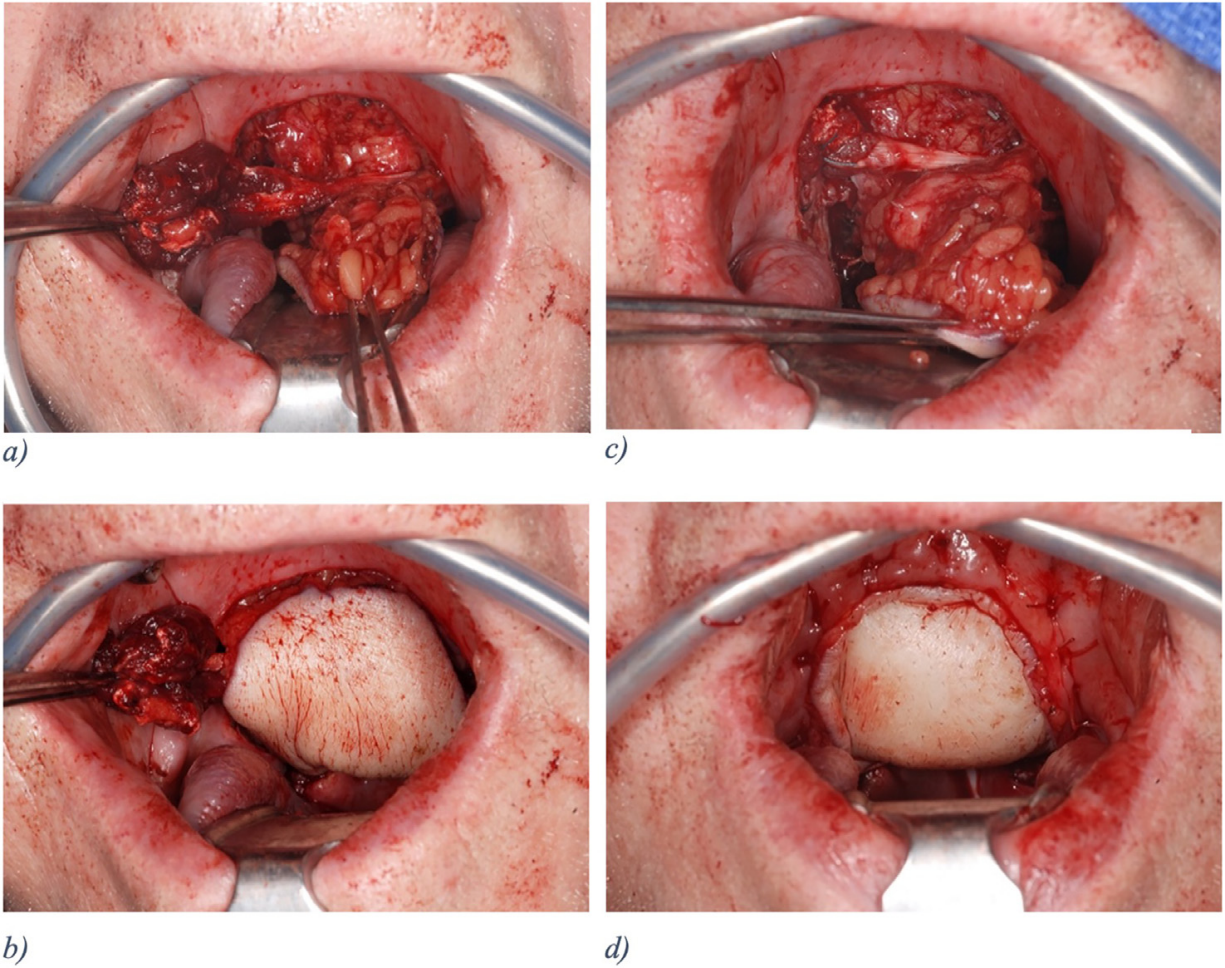
Intraoperative pictures showing the sandwich technique. **a.** Flap inset of the posterior wall of the radial forearm free flap (RFFF) inside out and the transposed digastric muscle. **b** and **c.** Digastric muscle lying between the posterior and anterior wall of the folded RFFF. **d.** Sutured anterior wall of the RFFF to the remnant borders of the defect zone to close the sandwich.

Afterward, the radialis flap was folded around the digastric muscle, placing the fascial aspect of the flap in contact with the muscle and keeping the epidermis on the external (Figure 7), creating a three-layered sandwich (digastric muscle between back and front part of the folded radialis flap) (Figure 8).

In cases of total or subtotal palatectomy, the procedure can be performed with bilateral muscle transfer and suturing of the digastric tendons in a crisscross manner to the corresponding contralateral remnants of the soft palate.

Microsurgical anastomosis of the RFFF was then performed to the neck vessels (e.g. artery end-to-end to the superior thyroid artery and veins end-to-side to the internal jugular vein with Polyamide 6 9-0 (Ethilone, Ethicon, Raritan, New Jersey, US). A nasogastric tube was placed and one to two easy-flow drains were always inserted in the neck.

The donor side of the radialis flap was closed by direct suture or skin graft. In the case of skin graft, vacuum therapy with a negative pressure of −125 mmHg was used to assist skin graft uptake.

### Postoperative management

The patients were transferred postoperatively to our intensive care unit for at least 24 hours. They received heparin 10,000 IU (Heparin Bichsel, Unterseen, Switzerland), 6 hours postoperatively, acetylsalycilic acid 300 mg orally (Aspirin, Bayer, Leverkusen, Germany) on the first postoperative day, and then acetylsalycilic acid 100 mg orally for 3 weeks. Flap control was done clinically (recapillarization, Doppler, color, temperature) and performed every hour during the first 24 hours, every 2 hours on the second postoperative day, every 4 hours on the third day, and every 8 hours from the fourth day on. For the skin graft at the donor site, the vacuum device was removed after 5 days.

Alimentation was administered through a percutaneous endoscopic gastrostomy (PEG) feeding tube for at least 2 weeks after the operation. In the case of favorable healing with complete recovery of the flap, alimentation could be restarted initially with liquids and afterward with a soft food diet. Depending on the clinical condition of the enoral and pharyngeal swelling, the tracheal cannula could be removed after 2 to 5 days.

## Results

### Cadaveric study

**Figure 9:**
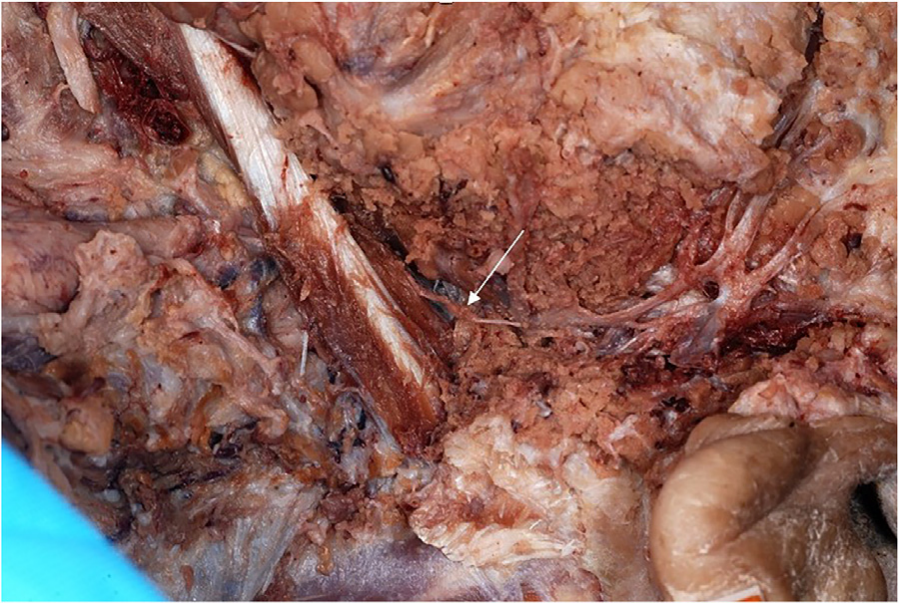
Anatomical study showing the independent innervation of the posterior belly of the digastric muscle from behind. Arrow indicates ramus digastricus coming from the facial nerve.

**Figure 10:**
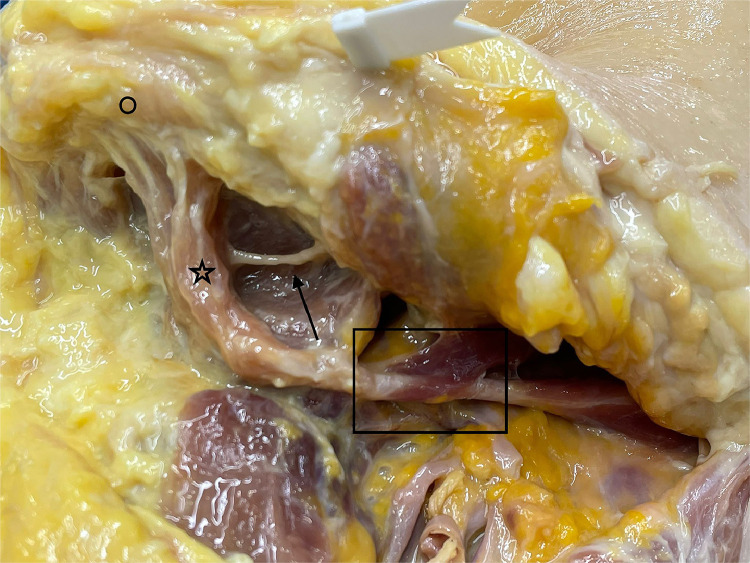
Separate innervation of anterior belly of digastric muscle, view from lateral on submental region. Arrow indicates mylohyoid nerve, star indicates anterior belly of digastric muscle, circle indicates submental, and rectangle is showing stylohyoid muscle surrounding intermediate tendon of digastric muscle.

Our cadaveric study with three cadavers (two females, one male) confirmed the separate innervation of the two digastric muscle bellies (arrows in Figures 9 and 10), which is crucial to keep the posterior venter muscle belly functional in our surgical technique.

With formaldehyde-preserved cadavers, we were not able to present the tiny branches innervating the two muscle bellies, but switching to Thiel fixation kept the tissue soft and allowed a more realistic surgical preparation.

**Figure 11:**
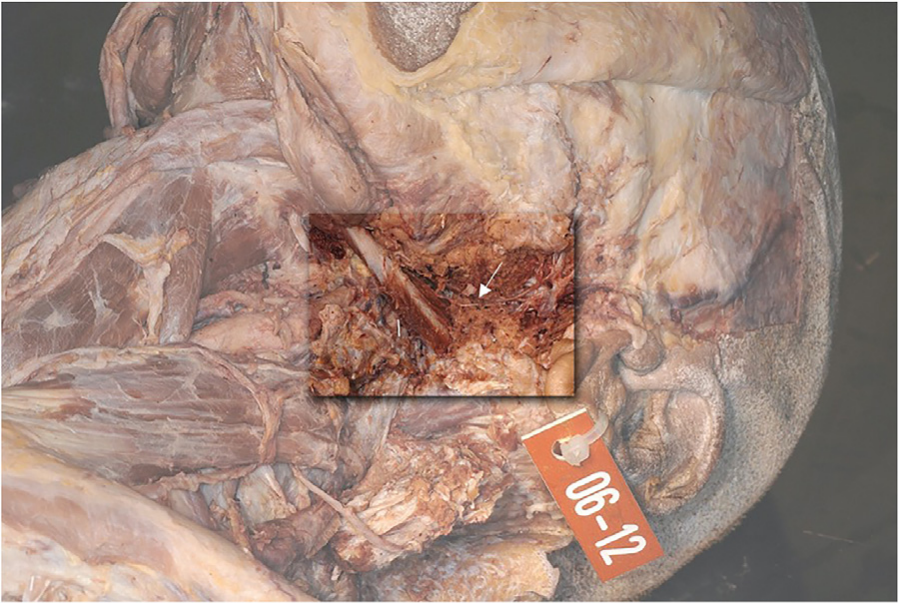
Anatomical study with view of the posterior belly of the digastric muscle and its separate innervation (arrow).

The anterior belly of the digastric muscle is innervated by the mylohyoid nerve (arrow Figure 10), which is a branch of the mandibular division of the trigeminal nerve. The posterior belly of the digastric muscle is innervated by the facial nerve through the ramus digastricus (arrow Figure 9, Figure 11).

**Figure 12:**
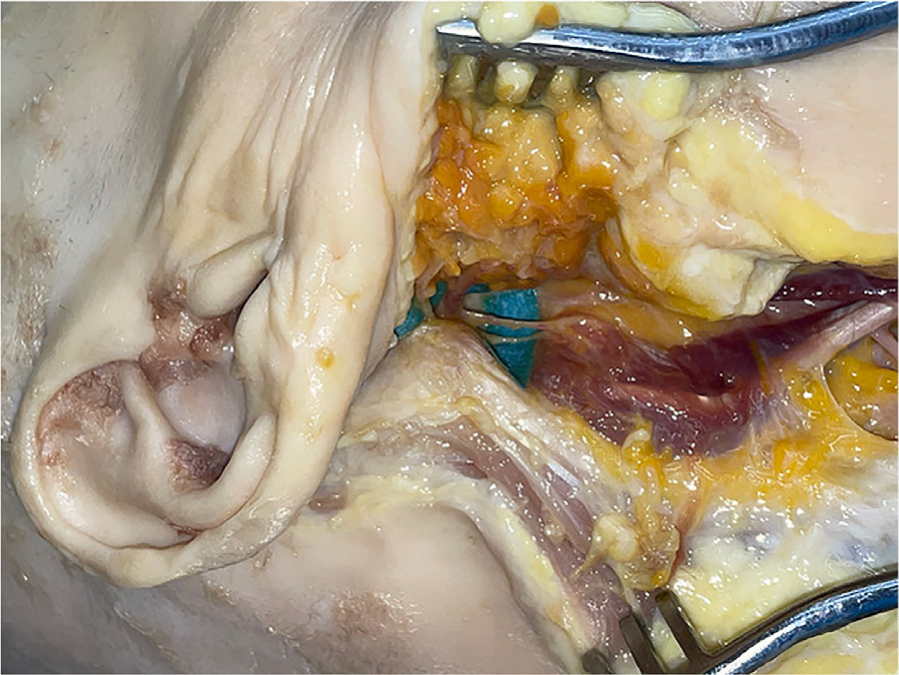
View from lateral on the nerve and artery entering the posterior belly from behind.

In the dissected three cadavers, the ramus digastricus entered the posterior belly from behind and medially. During a digastric transfer, this structure is typically protected and is therefore normally not visible during the operation (Figure 12).

During the removal of the jugulodigastric lymph nodes in Level IIa, the nerve might be at risk if the dissection goes under the level of the muscle in the posterior part.

The entry point from the facial nerve into the muscle was about 1,5–2,5 cm (Table 1). We could observe that the artery going into the muscle lied directly next to the nerve (Figure 12).

The mylohyoid nerve running on the inner side of the mandible entered the anterior belly from underneath (arrow, Figure 10).

The insertion of the anterior belly (star, Figure 10) was cut sharply with its nerve from its origin at the mandible (circle, Figure 10) and freed at the hyoid bone by opening the tendon loop (rectangle, Figure 10) and guiding the digastric muscle through the split tendons of the stylohyoid muscle.

**Figure 13:**
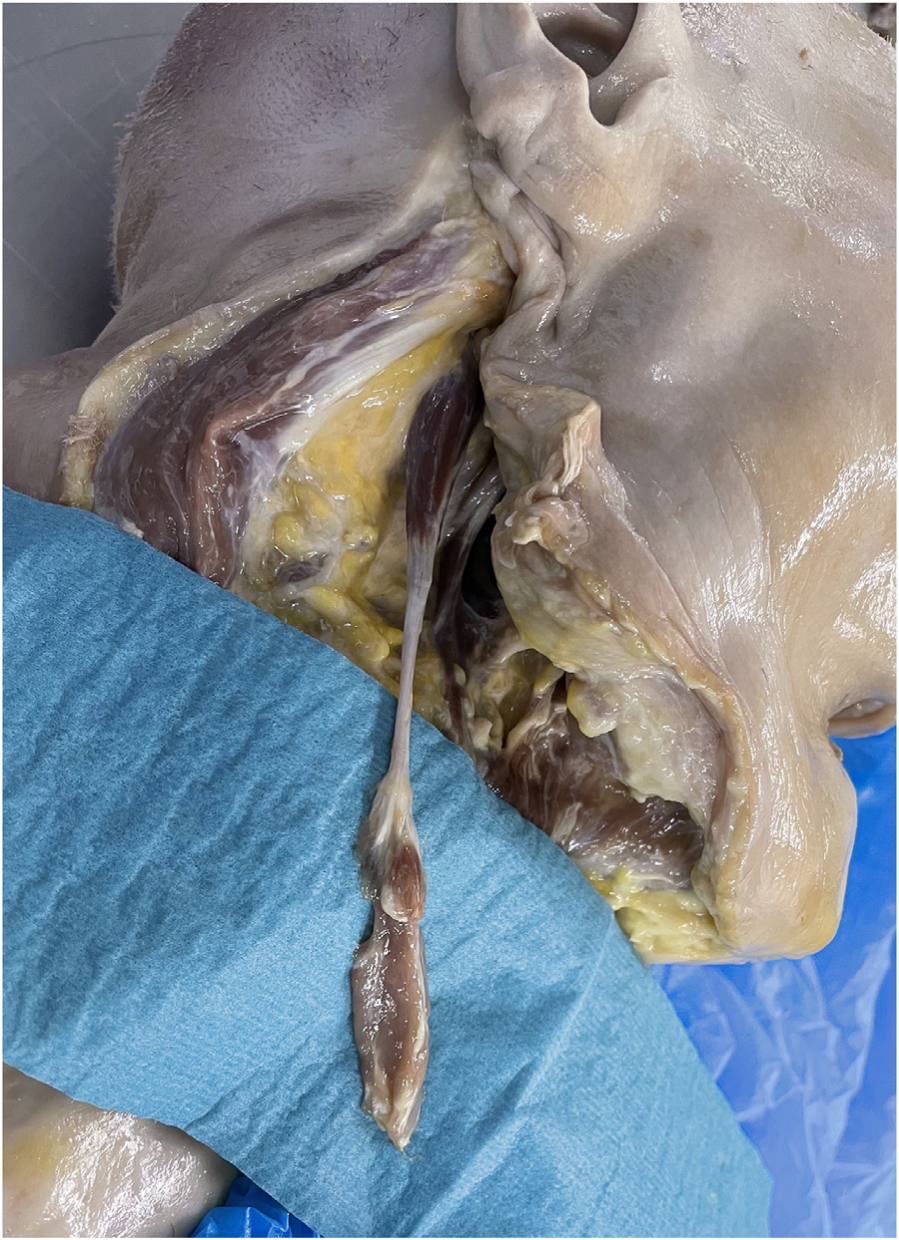
Mobilized anterior muscle belly before transfer.

The mobilized digastric muscle was then long enough (Figure 13) to reach the contralateral side of the soft palate with the anterior belly of the muscle. As important landmarks for the measurements, we identified the digastric ridge of the mastoid process for the posterior belly and the digastric fossa of the mandible which are the origins. The fibrous loop of the hyoid bone creates the junction zone of the two muscle bellies and was also included in our measurements. The length from mastoid to fibrous loop was between 7.5 and 8 cm and from the mandible to the fibrous loop 5.5 and 6.5 cm, which gave a total muscle distance of 13–14.5 cm (Table 1).

**Figure 14:**
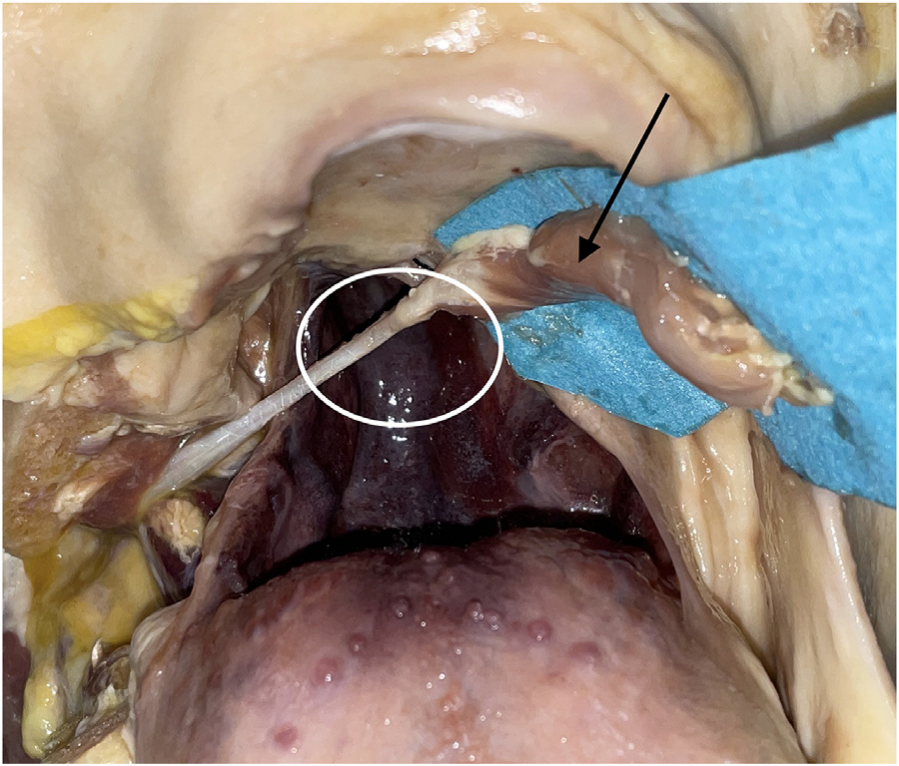
View from anterior into the oral cavity after muscle transfer (part of mandible removed for demonstration): White circle is showing defect of soft palate and arrow is showing anterior belly of digastric muscle.

**Figure 15:**
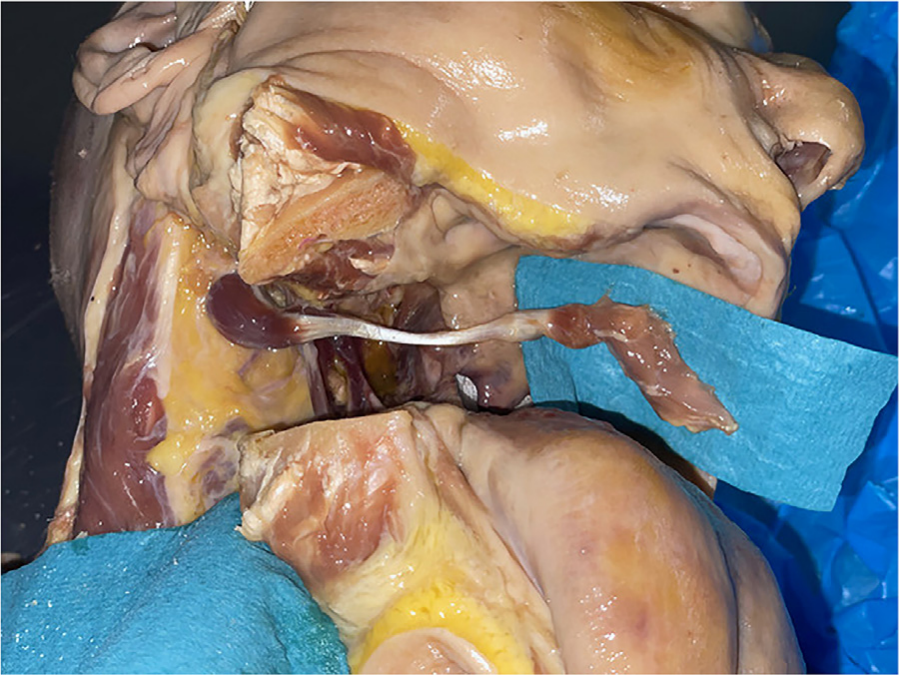
View from anterior right oblique after muscle transfer (part of mandible removed for demonstration) showing anterior and posterior muscle bellies with the intermediate tendon.

The muscle was then transferred into the oral cavity to the contralateral side of the soft palate passing the stylohyoid muscle ventrally (Figure 14, Figure 15). The length from the origin of the posterior belly to the base of the uvula was between 8.5 and 9 cm (Table 1); therefore, our transferred muscle had a remaining length of at least 4 cm. This excess length can be used for fixation.

Our cadaveric study proved the anatomical feasibility of the technique, forming a basis for implementing the technique in the clinical setting.

### Soft palate reconstruction

A total of eight patients were included in this retrospective review. Two patients were females and six were males, with a mean age of 56 years, ranging between 43 and 69 years.

All patients were affected by oropharynx carcinoma (stage T1-T3) infiltrating the soft palate. Regarding UICC stadium, five patients were classified as stadium IVa, two as stadium III, and one as stadium II. For TNM classification, three patients were identified as pT3, three as pT2, and two as pT1. The defect size ranged from two- thirds of the soft palate in five cases to complete in two cases; in one case, only half of the soft palate was involved. Risk factors were present in six of the eight patients, with four patients diagnosed with alcohol and nicotine abuse. Chronic obstructive pulmonary disease was present in one patient and cardiomyopathy in another. Four patients had arterial hypertension (Table 2).

**Table 1:** Important landmarks and measurements.

**Distances**	Cadaver 1 (female)	Cadaver 2 (female)	Cadaver 3 (male)
Origin posterior belly - base of the uvula	8.5 cm	9 cm	9 cm
Fibrous loop of hyoid bone - base of uvula	7.5 cm	8 cm	9 cm
Origin posterior belly - fibrous loop	7.5 cm	7.5 cm	8 cm
Origin anterior belly - fibrous loop	6 cm	5.5 cm	6.5 cm
Length of posterior + anterior belly	13.5 cm	13 cm	14.5 cm
Entry point R. digastricus in muscle	1.5 cm	2 cm	2.4 cm

R., Ramus

One patient received neoadjuvant radiochemotherapy before surgery; three patients received adjuvant radiochemotherapy and two received radiotherapy alone after surgery; and two patients had no adjuvant therapy, one of whom refused any further therapy.

Regarding complications, all eight patients showed no hematoma and none had problems with seroma or wound infection; in four patients, dehiscence of the RFFF occurred, but there was no single case in which a partial or full necrosis of the flap arose. Four of the eight patients had no complications.

Follow-up showed that swallowing of fluid and eating a soft diet was possible in seven of eight patients. Three of the eight patients were able to eat solid food without documented problems of velopharyngeal insufficiency; in two cases, additional long-term nutrition via a PEG (percutaneous endoscopic gastrostomy) feeding tube was necessary. In one of these two patients, swallowing problems occurred after adjuvant radiotherapy; the other patient had mucositis and dysphagia. Only one patient was not able to eat at least a soft diet because of aspiration that started after radiotherapy.

Regarding the speech outcome, all patients were able to carry on a conversation in an understandable manner with low nasalance. (Table 3).

**Table 2:** Patient data and complications.

Age (years)	Diagnosis	TNM	Stadium (UICC)	Soft palate defect size	Hematoma	Seroma	Infected wound	Flap dehiscence	Flap necrosis
60	Oropharyngeal squamous cell carcinoma left	pT2, pN2b (5/16), G3, V0, L0	IVa	2/3	No	No	No	Yes	No
48	Oropharyngeal squamous cell carcinoma right	pT3N0M0	III	2/3	No	No	No	No	No
61	Oropharyngeal squamous cell carcinoma left	pT3 pN0 (0/25) cM0 G2 R0	III	2/3	No	No	No	No	No
49	Oropharyngeal squamous cell carcinoma left	pT1 (m), pN2c (left 4/26; right 2/23), cM0, G3	IVa	Complete	No	No	No	No	No
61	Oropharyngeal squamous cell carcinoma right	pT2 pN2b (4/30) L1 V0 Pn0 G2	IVa	2/3	No	No	No	No	No
69	Oropharyngeal squamous cell carcinoma right	pT3, pN0 (0/29), L0, V0, Pn0 cM0 p 16+	IVa	2/3	No	No	No	Yes	No
59	Oropharyngeal squamous cell carcinoma left	pT1 pN2b G2	II	Complete	No	No	No	Yes	No
43	Oropharyngeal squamous cell carcinoma right	pT2 pN2b M0 G3 L1	IVa	1/2	No	No	No	Yes	No

TNM, TNM classification system of malignant tumors; UICC, Union for International Cancer Control staging system.

**Table 3:** Outcome of speech and diet, risk factors, and perioperative therapy.

Age (years)	Diet	Speech	Risk factor	Neoadjuvant therapy	Adjuvant therapy
60	Fluid and soft, partial via PEG	Intelligible, rhinophonia aperta	Alcohol, nicotine	No	RCT
48	Fluid and soft	Intelligible, little nasalance	Alcohol, nicotine, diabetes mellitus II	RCT	No
61	Only fluid, PEG because of aspiration after RT	Intelligible, nasalance after RT	COPD, hypertension	No	RT, palliative CT
49	Fluid, soft, and solid	Intelligible	Alcohol, nicotine, hypertension	No	RT
61	Fluid and soft	Intelligible, little nasalance	Hypertension, cardiomyopathy	No	RT
69	Fluid, soft, and solid	Intelligible, little nasalance	No	No	Refused
59	Fluid and soft	Intelligible	No	No	No
43	Fluid, soft, and solid	Intelligible	Alcohol, nicotine, hypertension	No	RCT

PEG, percutaneous endoscopic gastrostomy; RT, radiotherapy; COPD, chronic obstructive pulmonary disease; RCT, radiochemotherapy; CT, chemotherapy.

## Discussion

Our surgical concept involves the transposition of a muscle that is naturally involved in the physiological swallowing mechanism. It allows functional reconstruction without the need for postoperative relearning by the patient.

The sacrifice of the anterior belly of the digastric muscle does not seem to be problematical because it is not a key muscle in the swallowing process, as Okada et al.^14^ showed in their study. Because of the double separate and independent innervation of the digastric muscle, the posterior belly keeps its innervation despite muscle transposition. Through our cadaveric study, we confirmed this important anatomical feature. The technique is convenient to perform after neck dissection because the muscle has already been accessed and therefore needs additional operating time of only about 10 minutes. Moreover, this technique has a low complication rate in an already vulnerable patient collective.

Many different reconstruction techniques have been described in the literature to date. In 2017, Hamahata et al.^15^ described a reconstruction method of soft palate defects of more than two- thirds by using a folded anterolateral thigh flap, the results being superior to those found with their conventional method. After 1 year, however, most of the patients showed a larger pharyngeal isthmus with worsening results, which might occur because of reduced swelling of the flap over time. It is difficult to anticipate the final volume if a thick flap is used. There is a thin line between satisfactory sealing of the pharynx and breathing problems if the cross-sectional dimension of the pharynx port is not correct. Penfold et al.^16^ showed this in their 1996 study with a combined folded RFFF and pharyngeal flap in which the cross-sectional area of the pharyngeal port had to be more than 20 mm^2^ to prevent hypernasality and nasal regurgitation, but not significantly less than 20 mm^2^ because of the potential for impaired nasal breathing or apnea. With our technique, the cross-sectional diameter of the pharyngeal port is variable due to the muscle that contracts only during active muscle contraction; therefore, our patients showed no breathing problems despite their good speech quality.

The direction of the pulling vector imitates the lost sphincter function and improves sealing during swallowing. Extensive cancer resection of the soft palate on one side also leads to a weakening of the contralateral side because the muscle no longer has a countermove. By suturing the digastric muscle on the side wall of the resected soft palate, we reconstructed the entire palatal arch. This enables the remaining soft palate muscle of the healthy side to contract (separation of oro- and nasopharynx during swallowing) and a physiological vector is created to move the soft palate in the right direction. This needs a fixation point on the contralateral side of the hard palate to reconstruct the inverted V-shaped arch; otherwise, the functional part of the reconstruction does not work. If the resection is wider, the muscle of both sides can be used to recreate the functional arch (Figure 14). The defect size itself is covered with the radialis flap which can be planned accordingly.

The innervation of the posterior belly through the facial nerve does not require any relearning because it already takes part in the physiological swallowing process. This leads to better functional outcomes compared with those for known static operation techniques. Although our patient collective was small and further studies with objective examinations need to be performed, our results showed that seven of eight patients had a satisfactory outcome with the ability to swallow liquid and eat at least a soft diet. Almost half of the total patients were able to eat solid food. The only person who was not able to eat a soft diet had aspiration problems after radiotherapy and, at the same time, worsened nasalance in his speech. We assume that the radiation changed the local tissue quality, which made the reconstruction rigid. The radiation may also have led to shrinkage of the tissue volume, which makes it difficult to achieve a proper seal.

Regarding speech outcome, all our patients were able to carry on a conversation in an understandable manner with little nasalance. This is crucial for a patient's quality of life and one of the five goals that van der Sloot^17^ described in his article on soft palate reconstructions (separation of the sinonasal and oral cavities, intelligible speech, effective swallowing, mastication, and cosmesis).

In general, the complication rate of our technique was low in the patient group. The most common complication was flap dehiscence, which occurred in four patients. One explanation is that two of the four patients were smokers and addicted to alcohol. The sacrifice of the anterior belly of the digastric muscle in the donor site does not seem to be problematical because other muscles have the same function^14^ and there is no need to make another skin incision to access the muscle.

Our study has potential limitations. The main limitations are the small number of patients and the retrospective nature of the study. In subsequent studies, more detailed functional examinations (e.g. formant analysis, swallowing tests) with more patients should be performed. The main goal in this study, however, was to show the feasibility of a dynamic reconstruction method in the field of a soft palate reconstruction.

## Conclusion

By using the digastric muscle transfer plus forearm free flap in the reconstruction of soft palate defects of more than 50%, we introduced a reliable method of functional soft palate reconstruction that can be performed simultaneously with oncological resection without the need of any relearning process. We believe that this new element should be included in a treatment algorithm for surgical intervention. We showed that our dynamic technique has a low complication rate in a vulnerable patient collective. Because this technique is now well established in our interdisciplinary head and neck reconstruction procedures, we plan a second study involving the functional examination of more patients.

## Declaration of competing interest

None.

## Appendix Supplementary materials

Image, video 1

Image, video 2

## Footnotes

Supplementary material associated with this article can be found, in the online version, at doi:10.1016/j.jpra.2024.11.016.
