# Supplementary material for: Functional soft palate reconstruction
Source: JPRAS Open. 2024 Dec 2;43:293–308. doi: 10.1016/j.jpra.2024.11.016 (PMC11732850; doi:10.1016/j.jpra.2024.11.016)
Supplement: Supplementary file 2 [file mmc2.docx]

Transposition of digastric muscle into oral cavity
